# Vocalization Characteristics of the Indo‐Pacific Humpback Dolphins ( *Sousa chinensis* ) in Xiamen Bay With Insights on Regional Differences

**DOI:** 10.1002/ece3.73095

**Published:** 2026-02-11

**Authors:** Xuming Peng, Zhiyuan Hua, Fuxing Wu, Fei Zhang, Weijie Fu, Yupeng Li, Chuang Zhang, Wenzhan Ou, Wenjie Xiang, Bing Zhou, Zhongchang Song, Yu Zhang

**Affiliations:** ^1^ Key Laboratory of Underwater Acoustic Communication and Marine Information Technology of the Ministry of Education, College of Ocean and Earth Sciences Xiamen University China; ^2^ Hanjiang National Laboratory Wuhan China; ^3^ Third Institute of Oceanography Ministry of Natural Resources Xiamen China; ^4^ College of Engineering Huaqiao University Quanzhou China; ^5^ Dongshan Swire Marine Station (D‐SMART) Xiamen University Zhangzhou China; ^6^ National Observation and Research Station for the Taiwan Strait Marine Ecosystem Xiamen University Zhangzhou China

**Keywords:** acoustic characteristics, anthropogenic noise, Indo‐Pacific humpback dolphins, vessel traffic, vocalizations

## Abstract

Although whistles and high‐frequency clicks of Indo‐Pacific humpback dolphins ( *Sousa chinensis* ) had been documented in many wild populations, the small population inhabiting Xiamen Bay has received limited attention. Monthly surveys from 2021 to 2024 recorded a total of 107 whistles and 33,038 high‐quality clicks. Whistles exhibited mean minimum and maximum frequencies of 5.2 ± 2.2 kHz and 7.5 ± 2.7 kHz, respectively, with a mean duration of 247.6 ± 174.2 ms. Clicks had a peak frequency of 86.4 ± 19.5 kHz, a −3 dB bandwidth of 53.3 ± 14.6 kHz, and a duration of 22.3 ± 6.4 μs. Statistically, clicks from dolphins in the West sub‐region had a higher mean peak frequency, broader −3 dB bandwidth, and shorter duration than those from the East sub‐region, suggesting adaptations to local environmental conditions and vessel noise. No significant difference was observed in whistles between the two communities. These findings indicated a potential risk of population subdivision for the Xiamen Bay population, underscoring the urgency of protective measures that sustain connectivity and reduce anthropogenic pressures.

## Graphical abstract

The Indo‐Pacific humpback dolphin population in Xiamen Bay was divided into two geographically separated but socially associated communities inhabiting the West and East sub‐regions. Based on 3 years of boat‐based surveys, this study established baseline acoustic characteristics and investigated whether vocal patterns varied with habitat conditions between these two communities. Our findings reveal emerging inter‐community vocal divergence, highlighting the urgency of protective measures to mitigate anthropogenic pressures and reduce the risk of further fragmentation.

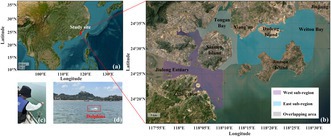

## 1 Introduction

Sound production and perception are fundamental capabilities that enable many aquatic animals to meet essential survival demands (Croll et al. 1999; Duarte et al. 2021; Fine and Parmentier 2015). These abilities are observed across a wide range of body sizes, from centimeter‐scale organisms such as snapping shrimp (Au and Banks 1998; Song et al. 2021) to some of the largest marine species, including blue whales (Schall et al. 2020). Acoustic signals serve as a key means for animals to obtain information from their environment, facilitating communication, navigation, prey detection, defense, and mate attraction (Amorim and Hawkins 2000; Au 1993; Cusano et al. 2021; Edds‐Walton 1997; Ladich 2019; Liu et al. 2013; Payne and Webb 1971; Popper et al. 2001). Among marine taxa, odontocetes are widely recognized as acoustic specialists because of their remarkable ability to integrate sound production and reception into a highly effective biosonar system (Au 1993; Busnel 2013). Dolphin biosonar outperforms man‐made sonar in many respects, particularly in its flexibility to modulate sound characteristics and beam patterns in response to changing environments, which has drawn substantial scientific interest in their vocal behaviors under various ecological contexts (Au 1993; Madsen et al. 2023; Moss et al. 2023; Wisniewska et al. 2015).

Odontocetes produce a diverse repertoire of sounds such as whistles, clicks, and burst‐pulse signals (Au 1993; Janik and Sayigh 2013; Jones et al. 2020; Lammers et al. 2004; Madsen and Wahlberg 2007; Martin et al. 2019). Whistles are frequency‐modulated signals primarily used for social communication which typically have fundamental frequencies below 20 kHz with harmonics extending above 100 kHz (Janik and Sayigh 2013; Jones et al. 2020; Thomsen et al. 2001; Wang et al. 2013). Short, broadband clicks are essential for echolocation and navigation, particularly in *Delphinidae*, which can produce whistles for communication (Janik and Sayigh 2013). In species such as sperm whales that do not produce whistles, clicks serve both navigational and communicative functions (Andreas et al. 2022; Oliveira et al. 2013). Burst‐pulse sounds consist of rapid click trains used in close‐range and intraspecific communication, as documented in Heaviside's dolphins ( *Cephalorhynchus heavisidii* ), Commerson's dolphins ( *Cephalorhynchus commersonii* ), and harbor porpoises ( *Phocoena phocoena* ) (Martin et al. 2018; Sørensen et al. 2023; Yoshida et al. 2014). These acoustic behaviors are essential for survival in dark or turbid marine habitats and are shaped by both natural conditions and anthropogenic disturbances, with individuals of the same species often showing variation in vocal characteristics under different environmental settings and levels of human‐induced noise (Buckstaff 2004; De Vere et al. 2018; Holt et al. 2009; Bhagarathi et al. 2024; Popper and Hawkins 2016; Scheifele et al. 2005). Consequently, examining vocal behaviors in populations of the same species inhabiting different environments represents an important research direction, offering insights relevant to species conservation and the management of acoustically sensitive marine ecosystems.

The Indo‐Pacific humpback dolphin ( *Sousa chinensis* ) is a medium‐sized odontocete distributed along China's coastal waters and extending into the eastern Indian Ocean, and is currently classified as Vulnerable on the International Union for Conservation of Nature (IUCN) Red List (Chen et al. 2018; Jefferson and Smith 2016; Shirihai et al. 2006; Wang et al. 2022). In Chinese waters, the species is primarily found in the southeast, with several local populations, including those in the Pearl River Estuary (PRE), Xiamen Bay, Leizhou Bay, Beibu Gulf, western Taiwan, and western Hainan (Chen et al. 2009, 2010; Li et al. 2015; Wang et al. 2021). Among these, the Xiamen Bay population is of particular concern, as it has experienced marked declines over the past two decades due to intense anthropogenic pressures (Wang et al. 2018; Wu et al. 2014, 2025; Zeng et al. 2020). Habitat degradation caused by coastal development and severe pollution has left this population critically endangered (Lu et al. 2024; Wang et al. 2018; Zeng et al. 2020), while prolonged exposure to anthropogenic noise further reduces communication efficiency and coordination among dolphin groups, decreasing task success rates by up to 22.5% (Sørensen et al. 2023).

A comprehensive understanding of the acoustic behavior of the Xiamen Bay population is therefore crucial for effective habitat management and species conservation. However, studies of its acoustic repertoire remain limited, with only a few investigations into whistles and clicks reported to date (Niu et al. 2012; Wang, Xu, et al. 2014; Zhang et al. 2025). Using average‐linkage hierarchical cluster analysis based on the Half‐Weight Index (HWI), previous photo‐identification surveys suggested that this population was divided into two geographically separated and statistically distinct communities, located in the West and East sub‐regions of Xiamen Bay (Wang et al. 2015). The East sub‐region is characterized by shallow flats with sandy substrates and some areas used as marine aquaculture zones, whereas the West sub‐region is dominated by estuarine processes and characterized by intense vessel traffic. In fission‐fusion societies like *S. chinensis* , the environmental constraints can drive fine‐scale vocal variation, representing significant functional adaptations and highlighting the risk of potential fragmentation. However, it remains unclear whether their vocal repertoires also differ.

In this study, we investigated the acoustic behavior of *S. chinensis* in Xiamen Bay through long‐term boat‐based field surveys conducted over 3 years. We recorded both whistles and clicks to establish baseline acoustic characteristics for this population. Furthermore, by comparing the acoustic features of dolphins in the West and East sub‐regions, we examined whether geographic separation and varying levels of anthropogenic disturbance influence vocal performance in adjacent groups inhabiting the same broader habitat.

## 2 Materials and Methods

### 2.1 Field Survey

**FIGURE 1:**
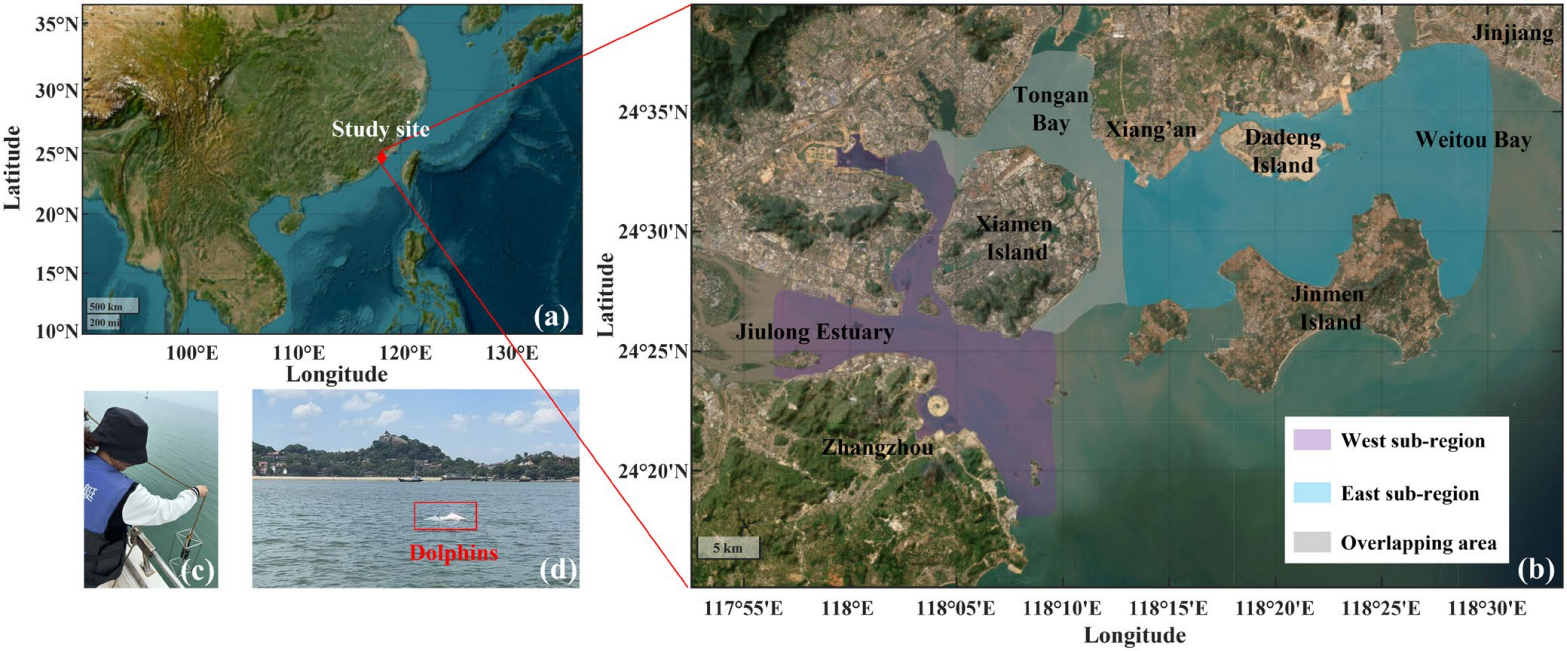
(a) An overall view of the location of the study site in a regional context. (b) Enlarged map of the study location. (c) Diagram of the on‐site survey recording. (d) Indo‐Pacific humpback dolphins encountered during a boat‐based survey.

From June 2021 to September 2024, monthly boat‐based surveys were conducted in Xiamen Bay, southeastern China, covering a 750 km^2^ study area (Figure 1). The study area was divided into three sub‐regions: West sub‐region (including Western Harbor and Jiulong River Estuary), East sub‐region (including the Dadeng‐Xiaodeng region and Weitou Bay), and an overlapping zone (Wang et al. 2015; Wu et al. 2025; Zeng et al. 2020).

Surveys were conducted using a 20 m vessel equipped with a 50‐hp inboard motor, traveling at 10–13 km/h under calm sea conditions (Beaufort sea state < 3). Observers were stationed on the upper deck (3.5 m above the water surface) to photograph dolphins for subsequent identification, estimate group size, and document behavioral states. Acoustic recordings were initiated immediately upon dolphin encounters using a SoundTrap ST300HF recorder (Ocean Instruments Ltd., New Zealand), comprising a power module, an analog‐to‐digital converter, and 256 GB of onboard storage, with a linear frequency range of 20 Hz to 150 kHz. To ensure high‐fidelity data collection, the sampling rate was set at 576 kHz, yielding a Nyquist frequency of 288 kHz, sufficient to capture the full frequency range of *S. chinensis* clicks, which falls well below 150 kHz. The recorder was secured to a custom‐made stainless‐steel bracket (150 × 150 × 400 mm) using two screws and deployed at a depth of 2.0 m, with slight fluctuations caused by water flow. The design composed of stainless‐steel bars ensured the hydrophone remained exposed to the seawater during the recording period to collect signals continuously. During deployments, the vessel's engine was turned off to minimize noise interference. The effective recording time was defined as the period when the SoundTrap remained submerged.

### 2.2 Acoustic Analysis on Clicks

To begin, a high‐pass filter was applied to eliminate low‐frequency ocean noise below 5 kHz. Clicks were short and broadband signals that resemble Gabor wavelets, and thus were initially detected using the Teager‐Kaiser Energy Operator (TKEO) detector (Kandia and Stylianou 2006; Luo et al. 2019; Madhusudhana et al. 2015):

(1) $$\Psi \left[X_{n}\right]=X_{n}^{2}-X_{n-1}X_{n+1}$$

where *X*_*n*_ represents the signal. The TKEO output was then filtered using a Gaussian filter and a rectangular filter with identical gain and length to highlight click candidates. The output of the Gaussian filter MAF_1_ is expressed as:

(2) $$\begin{array}{c} {\mathrm{MAF}}_{1}\left(n\right)=\frac{T_{S}}{\sigma_{G}\sqrt{2\pi }}e^{-\frac{{\left(nT_{S}\right)}^{2}}{2{\sigma_{G}}^{2}}}\; \end{array}$$

where *n* = *−N, …, −2, −1,0,1,2,…, N*, corresponding to the index of sample points in the filter and $\sigma_{G}=\text{FWHM}/4\sqrt{\ln\left(2\right)}$ denotes the standard deviation of the Gaussian function, with *FWHM* indicating the width of the Gaussian filter at half its peak amplitude. *T*_*s*_ is the sampling interval. The rectangular filter was employed to smooth the TKEO output and obtain the average energy over a specified time window. The rectangular filter MAF_2_ is defined as:

(3) $$\begin{array}{c} {\mathrm{MAF}}_{2}\left(n\right)=\frac{\sum_{m=-N}^{N}{\mathrm{MAF}}_{1}\left(m\right)}{2N+1}\; \end{array}$$

The Filter Difference Ratio (FDR) can be used to detect potential click pulses, which is defined as:

(4) $$\begin{array}{c} \mathrm{FDR}=\frac{h_{{\mathrm{MAF}}_{1}\left(n\right)}-h_{{\mathrm{MAF}}_{2}\left(n\right)}}{h_{{\mathrm{MAF}}_{1}\left(n\right)}}\; \end{array}$$

where $h_{{\mathrm{MAF}}_{1}\left(n\right)}$ and $h_{{\mathrm{MAF}}_{2}\left(n\right)}$ represent the outputs of the TKEO signal after Gaussian filtering and rectangular filtering, respectively. Locations with FDR values exceeding the threshold are identified as potential signals. In this study, the *FWHM* and *FDR* threshold were set to 0.10 ms and 0.7, respectively.

**FIGURE 2:**
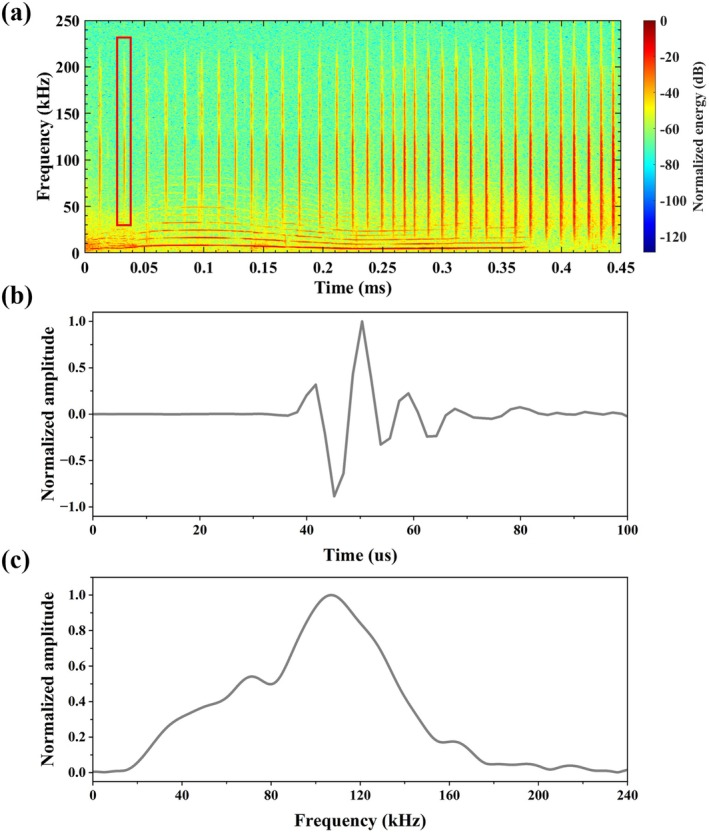
(a) The spectrogram of an example click train. (b) The time series of a click extracted from the red box in (a) and (c) its corresponding spectra.

The detector output typically contained both target clicks and other broadband signals such as snapping shrimp pulses. To accurately identify dolphin clicks from candidate pulses, we developed a backpropagation (BP) neural network, achieving an average identification accuracy of 90.1%, an F1‐score of 0.898, and an AUC of 0.987. Many of the extracted clicks exhibited reflections following the main signal, so a strict selection protocol was applied to retain high‐quality clicks for subsequent analysis (Song et al. 2023). To isolate the principal component of each click, the signal peak was used as a reference point, and a variable number of neighboring data points were extracted depending on the click's temporal characteristics. The broadband nature of the clicks was evident in the spectral analysis (Figure 2). We then analyzed their acoustic characteristics, including peak frequency, duration, −3 dB bandwidth, −6 dB bandwidth, −10 dB bandwidth, and peak‐to‐peak sound pressure level following previous studies (Fang et al. 2015; Madsen and Wahlberg 2007).

### 2.3 Acoustic Analysis on Whistles

The detection of whistles was performed using a connected‐component algorithm to examine the whistles in time‐frequency representation (Sun et al. 2015). To begin, files were first segmented into frames to reduce analysis burden. A frame length of 65,792 samples was chosen with an overlap of 13,824 samples between adjacent frames. The signal was processed using the Short‐Time Fourier Transform (STFT):

(5) $$\begin{array}{c} F\left(m,f\right)=\sum_{n=-\infty }^{\infty }x\left[n\right]w\left[n-m\right]e^{-j2\mathrm{\pi fn}}\; \end{array}$$

where *w*[*n*] denotes the window and a Hanning window of length 1024 samples was employed. The time‐frequency representation was obtained through logarithmic transformation:

(6) $$\begin{array}{c} P\left(m,f\right)=20\log_{10}\left|F\left(m,f\right)\right|\; \end{array}$$

After obtaining the spectrogram, a 3 × 3 median filter was applied to filter isolate noise using the following formula:

(7) $$\begin{array}{c} P^{\prime }\left(m,f\right)=P\left(m,f\right)+3\times \mathrm{std}\left(P\right)-\bar{P}\; \end{array}$$

where $\mathrm{std}\left(P\right)$ and $\bar{P}$ represent the standard deviation and mean value of the spectrogram. Subsequently, adaptive whistle frequency contour extraction was performed using adaptive thresholding. For each pixel, the threshold was defined as the mean intensity within a circular neighborhood of radius 15. Pixels exceeding this threshold were retained, whereas those below it were set to zero. Connected component analysis was then applied to the binary spectrogram to identify continuous regions corresponding to whistle signals. To eliminate click interference, connected regions with horizontal lengths shorter than 11 pixels were discarded, as whistle signals typically exhibit longer durations along the time axis. Additionally, to remove noise interference, connected regions with maximum pixel values below 10 were identified as noise and discarded.

**FIGURE 3:**
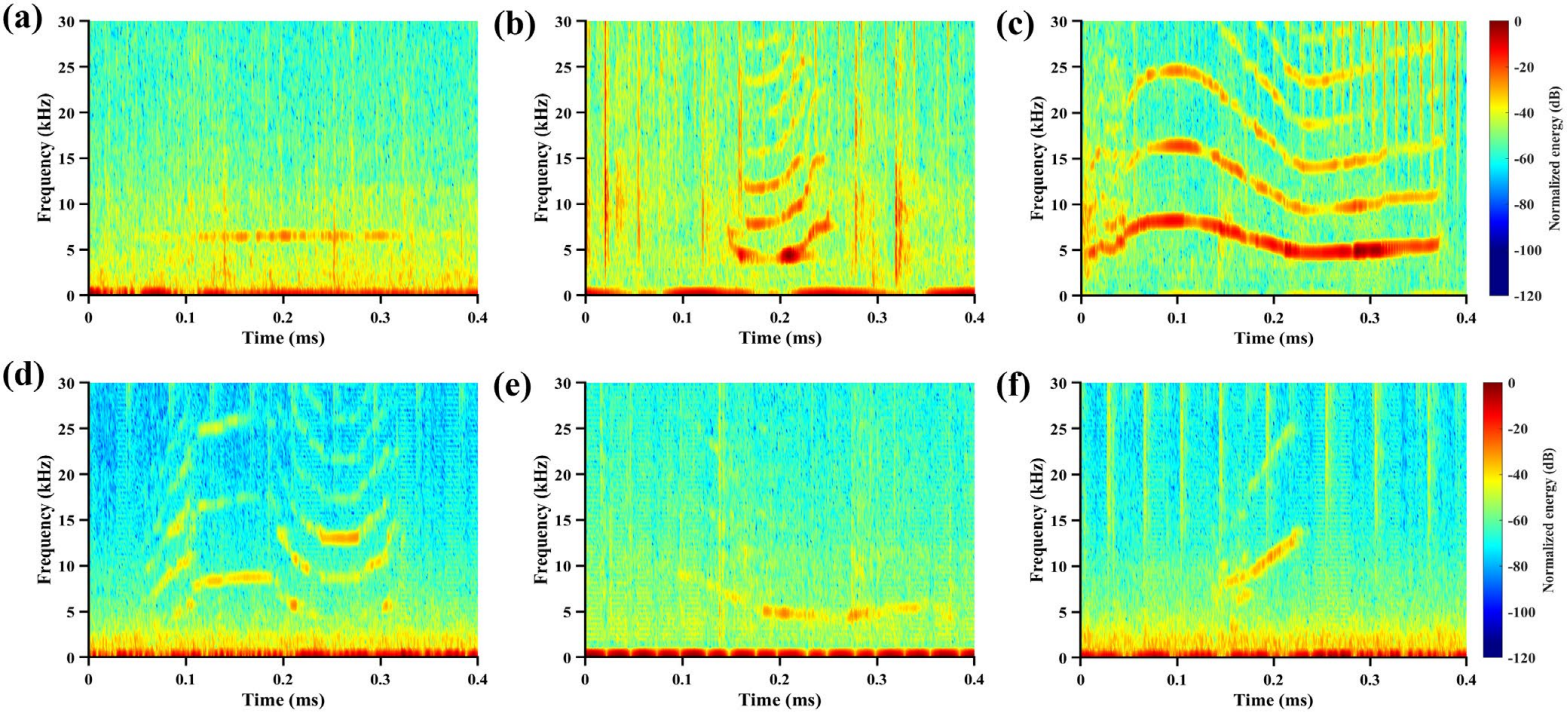
The spectrograms of examples of six types of whistles. (a) Constant‐frequency whistle. (b) Concave whistle. (c) Convex whistle. (d) Sinusoidal whistle. (e) Downsweep whistle. (f) Upsweep whistle.

Following automated extraction, all candidate whistle signals were manually inspected to ensure accuracy. The extracted whistles were classified into constant‐frequency, upsweep, downsweep, concave, convex, and sinusoidal types based on their frequency variation patterns in spectrograms (Figure 3) (Bazúa‐Durán and Au 2002; Wang et al. 2013). To better study the characteristics of dolphin whistle signals, this research calculated the parameters, including duration, beginning frequency, ending frequency, minimum frequency, maximum frequency, as well as frequency range defined as the difference between the maximum and minimum frequencies.

### 2.4 Comparison of Signals Between the West and East Sub‐Regions

We compared the vocalizations of dolphins inhabiting the West and East sub‐regions using statistical significance testing, with clicks and whistles classified according to the recording sites. The West and East sub‐regions covered 24.30°–24.57° N, 117.94°–118.16° E and 24.45°–24.63° N, 118.22°–118.50° E, respectively (Figure 1). Prior to comparative testing, normality assessment was performed on the datasets of vessel density, click, and whistle. Normality was evaluated using the Shapiro–Wilk test for datasets with small sample sizes (*n* ≤ 50) and the Kolmogorov–Smirnov test was used for datasets with large sample sizes (*n* > 50) (Yazici and Yolacan 2007). Given that the data were not normally distributed in this study, the non‐parametric two‐tailed Mann–Whitney U test was then applied to evaluate inter‐regional differences in acoustic characteristics.

## 3 Results

**FIGURE 4:**
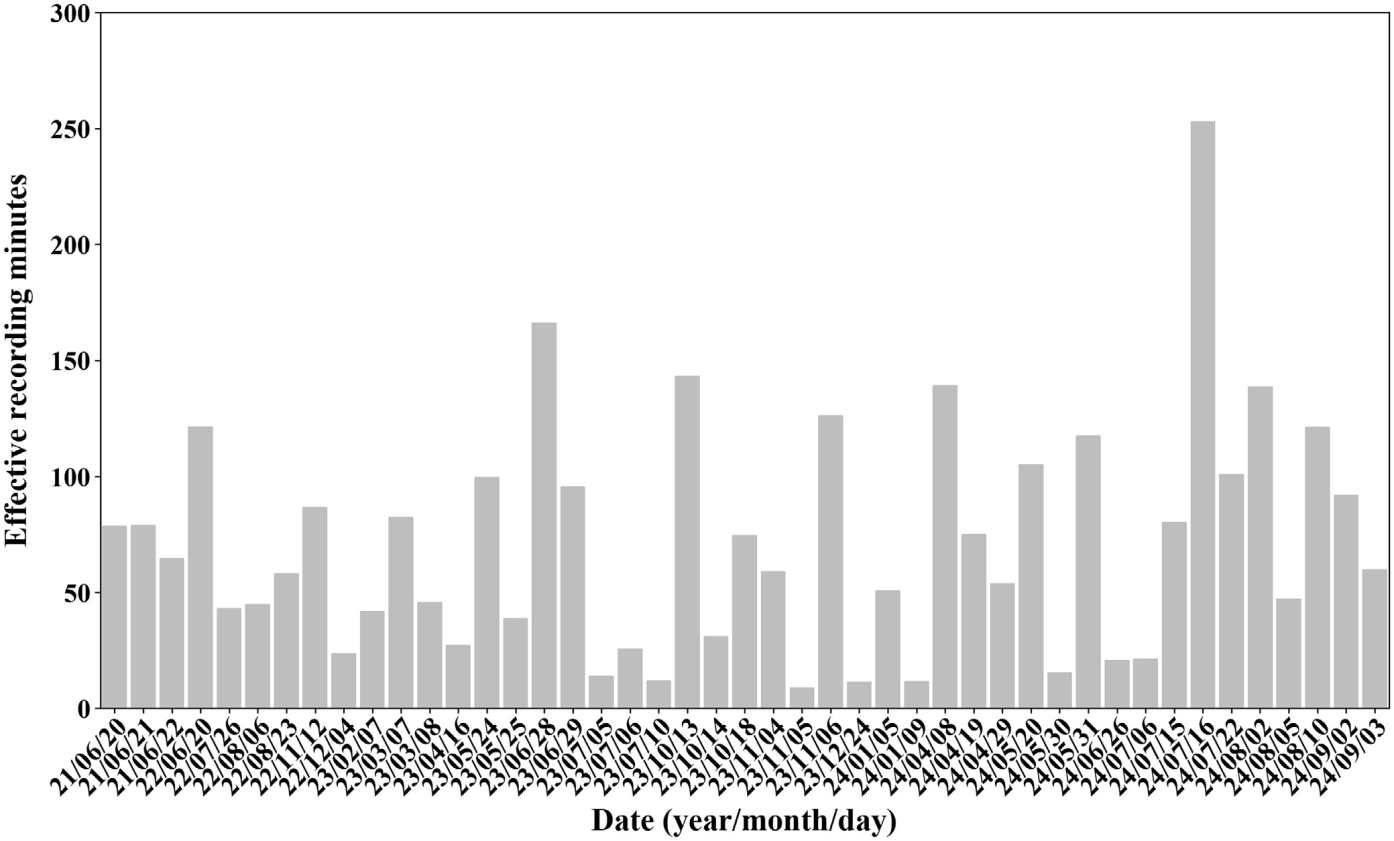
The effective recording minutes for 45 surveys.

Over the three‐year period, 45 acoustic monitoring surveys were conducted in Xiamen Bay, and dolphin clicks were successfully recorded in 43 of them. The average effective recording time per survey was 70.9 min, with values ranging from 8.6 to 252.7 min (Figure 4). During recordings, dolphins were observed in a variety of behavioral contexts, with group sizes ranging from 3 to 18 individuals. In total, 71,708 clicks were initially extracted. After removing low‐quality signals and those affected by reverberation, 33,038 high‐quality clicks were retained for analysis. During the 45 surveys, whistles were recorded in only 18 surveys, yielding 107 whistles in total.

### 3.1 Acoustic Parameter Statistics on Clicks and Whistles

The clicks of Indo‐Pacific humpback dolphins in Xiamen Bay exhibited short and broadband characteristics (Table 1). The duration of the clicks ranged from 6.9 to 48.6 μs, with a mean of 22.3 ± 6.4 μs. Peak frequency spanned 31.5–133.9 kHz, with an average of 86.4 ± 19.5 kHz, and the −3 dB bandwidth varied between 19.4 and 130.6 kHz, with a mean of 53.3 ± 14.6 kHz. The −6 dB and −10 dB bandwidth ranged from 30.1 to 169.7 kHz and 44.0 to 212.7 kHz, with mean values of 81.5 ± 18.9 kHz and 109.8 ± 23.3 kHz, respectively. The average peak‐to‐peak sound pressure level of the recorded clicks was 162.8 ± 9.5 dB.

**TABLE 1:** A summary of click characteristics where SD = standard deviation, f_p_ = peak frequency, BW_−3dB_ = −3 dB bandwidth, BW_−6dB_ = −6 dB bandwidth, BW_−10dB_ = −10 dB bandwidth and SPL_pp_ = peak‐to‐peak sound pressure level.

Acoustic parameters		Total (*n* = 33,038)	West (*n* = 24,033)	East (*n* = 9005)
f_p_ (kHz)	Mean ± SD	86.4 ± 19.5	86.8 ± 19.4	85.4 ± 19.8
	Range	31.5–133.9	31.5–131.6	32.0–133.9
BW_−3dB_ (kHz)	Mean ± SD	53.3 ± 14.6	53.7 ± 14.7	52.2 ± 14.3
	Range	19.4–130.6	19.4–130.6	19.6–113.9
BW_−6dB_ (kHz)	Mean ± SD	81.5 ± 18.9	82.3 ± 18.7	79.4 ± 19.1
	Range	30.1–169.7	30.1–169.7	33.5–168.7
BW_−10dB_ (kHz)	Mean ± SD	109.8 ± 23.3	110.2 ± 22.5	108.6 ± 25.3
	Range	44.0–212.7	44.0–209.6	48.2–212.7
Duration (μs)	Mean ± SD	22.3 ± 6.4	22.0 ± 6.2	23.1 ± 6.7
	Range	6.9–48.6	6.9–45.1	8.7–48.6
SPL_pp_ (dB re 1 μPa)	Mean ± SD	162.8 ± 9.5	163.6 ± 8.3	160.7 ± 11.9
	Range	123.8–193.2	126.8–193.2	123.8–187.0

**FIGURE 5:**
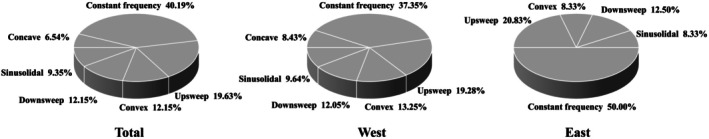
Pie chart of the proportion of different types of whistles for the total, East and West sub‐regions, respectively.

Out of the 107 recorded whistles, 83 were from the West sub‐region, accounting for about 77.6%. Constant‐frequency whistles were the predominant type in both sub‐regions, followed by upsweep whistles (Figure 5), whereas concave whistles occurred exclusively in the West sub‐region. Whistles of each type were aggregated for acoustic parameter analysis and an example for each type of whistle can be found in Figure 3. Overall, constant‐frequency whistles had a number of 43, accounting for the highest percentage (40.19%) among the six types, while only 7 concave whistles were found.

Descriptive statistics of whistle parameters are summarized in Table 2. The average whistle duration was 247.6 ± 174.2 ms and ranged from 21.4 to 937.0 ms, with the longest and shortest durations observed in constant‐frequency and concave whistles, respectively. Beginning frequency (BF) averaged 5.9 ± 2.4 kHz, slightly lower than the ending frequency (EF, 6.4 ± 2.8 kHz). Minimum frequency (MinF) and maximum frequency (MaxF) ranged from 2.2 to 12.9 kHz and 4.0 to 15.9 kHz, with mean values of 5.2 ± 2.2 kHz and 7.5 ± 2.7 kHz, respectively. The frequency range averaged 2.4 ± 2.1 kHz, spanning 0.1–10.5 kHz.

**TABLE 2:** A summary of whistle characteristics, where SD = standard deviation, BF = beginning frequency, EF = ending frequency, MinF = minimum frequency and MaxF = maximum frequency.

Acoustic parameters		Total (*n* = 107)	West (*n* = 83)	East (*n* = 24)
Duration (ms)	Mean ± SD	247.6 ± 174.2	246.8 ± 179.7	250.3 ± 157.3
	Range	21.4–937.0	21.4–937.0	25.1–642.1
BF (kHz)	Mean ± SD	5.9 ± 2.4	5.9 ± 2.2	6.1 ± 2.9
	Range	3.1–13.3	3.1–13.3	3.2–12.4
EF (kHz)	Mean ± SD	6.4 ± 2.8	6.4 ± 2.9	6.4 ± 2.6
	Range	2.4–15.9	2.4–15.9	4.1–11.6
MinF (kHz)	Mean ± SD	5.2 ± 2.2	5.1 ± 2.1	5.5 ± 2.8
	Range	2.2–12.9	2.4–12.9	2.2–12.4
MaxF (kHz)	Mean ± SD	7.5 ± 2.7	7.6 ± 2.8	7.1 ± 2.6
	Range	4.0–15.9	4.0–15.9	4.1–11.6
Frequency Range (kHz)	Mean ± SD	2.4 ± 2.1	2.6 ± 2.2	1.8 ± 1.3
	Range	0.1–10.5	0.1–10.5	0.4–5.1

### 3.2 Comparison of Clicks and Whistles Between the East and West Sub‐Regions

**FIGURE 6:**
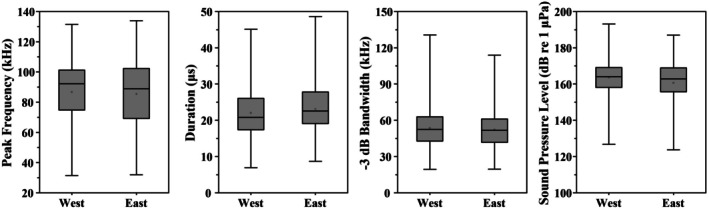
Comparison box plots of peak frequency, duration, −3 dB bandwidth and peak‐to‐peak sound pressure level for clicks between the East and West groups.

Significant differences in click acoustic parameters were observed between the West and East sub‐regions (Figure 6 and Table 1). The average peak frequency was 86.8 ± 19.4 kHz for the West group, significantly higher than 85.4 ± 19.8 kHz of the East group ($U=1.12\times 10^{8}$, *p* < 0.01). Click durations also differed significantly ($U=1.18\times 10^{8}$, *p* < 0.01), with 22.0 ± 6.2 μs and 23.1 ± 6.7 μs on average for the West and East sub‐regions, respectively. The −3 dB, −6 dB, and −10 dB bandwidths of the clicks recorded from the West sub‐region averaged 53.7 ± 14.7 kHz, 82.3 ± 18.7 kHz, and 110.2 ± 22.5 kHz, respectively, all significantly higher than 52.2 ± 14.3 kHz, 79.4 ± 19.1 kHz, and 108.6 ± 25.3 kHz in the East sub‐region ($U=1.14\times 10^{8}$, *p* < 0.01; $U=1.20\times 10^{8}$, *p* < 0.01; $U=1.14\times 10^{8}$, *p* < 0.01). Overall, dolphins inhabiting the East sub‐region produced clicks with longer durations, lower peak frequencies, and narrower frequency ranges. The average peak‐to‐peak sound pressure level (*SPL*_*pp*_) for the West group was 163.6 ± 8.3 dB, higher than 160.7 ± 11.9 dB for the East group ($U=1.19\times 10^{8}$, *p* < 0.01).

**FIGURE 7:**
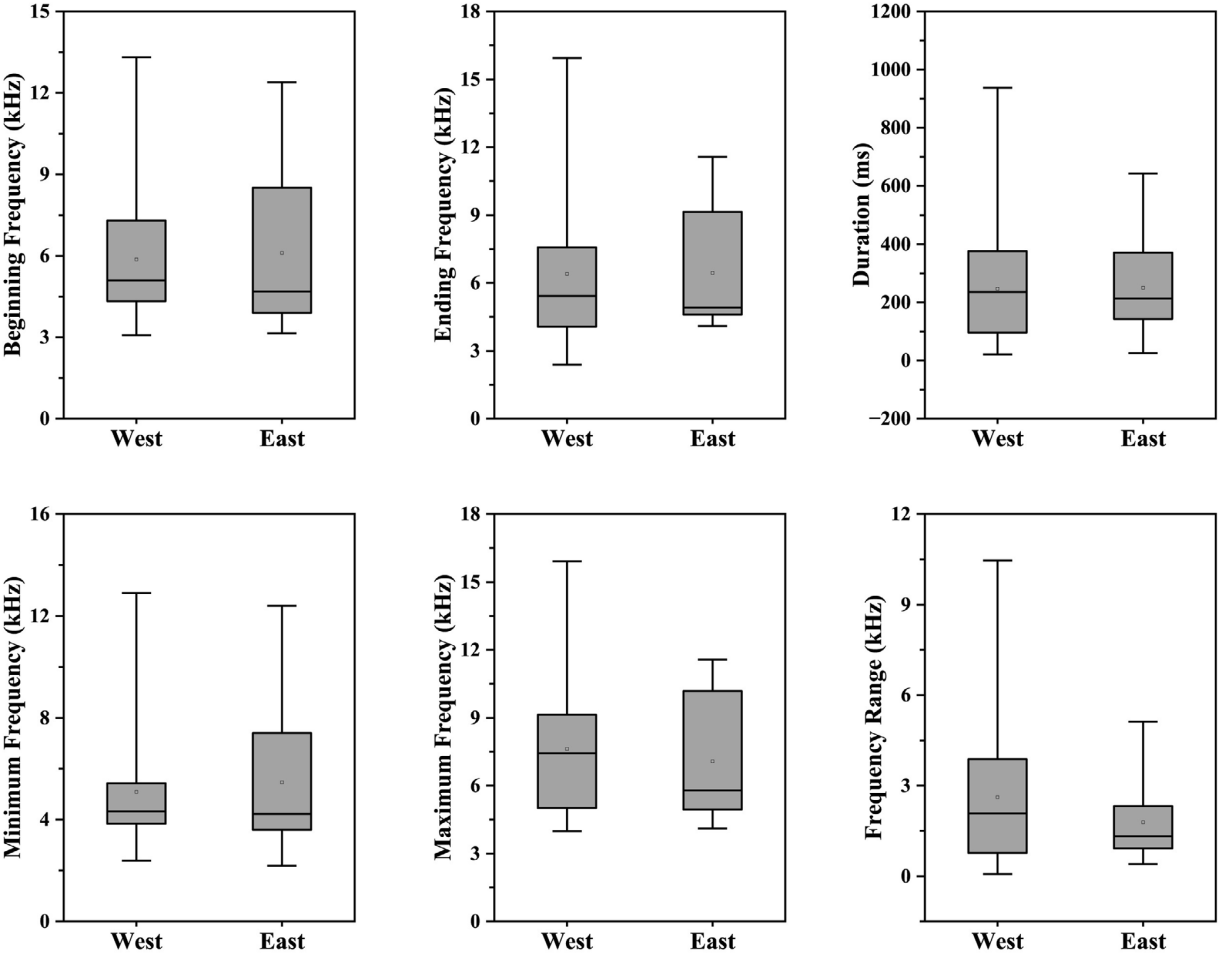
Comparison box plots of beginning frequency, ending frequency, duration, minimum frequency, maximum frequency, and frequency range for whistles recorded in the East and West sub‐regions.

With respect to whistle, the mean duration for the West group was 246.8 ± 179.7 ms, showing no significant difference from 250.3 ± 157.3 ms of the East group (Figure 7 and Table 2; U = 935, *p* = 0.65). Similarly, no significant inter‐regional differences were found in the beginning frequency, ending frequency, minimum frequency, maximum frequency, and frequency range of whistles (*p* > 0.05). In the West sub‐region, these parameters averaged 5.9 ± 2.2 kHz, 6.4 ± 2.9 kHz, 5.1 ± 2.1 kHz, 7.6 ± 2.8 kHz, and 2.6 ± 2.2 kHz, respectively. Corresponding values were 6.1 ± 2.9 kHz, 6.4 ± 2.6 kHz, 5.5 ± 2.8 kHz, 7.1 ± 2.6 kHz, and 1.8 ± 1.3 kHz in the East sub‐region.

## 4 Discussion

The acoustic signals of Indo‐Pacific humpback dolphins have been reported in various studies (Fang et al. 2015; Goold and Jefferson 2004; Niu et al. 2012; Van Parijs and Corkeron 2001; Wang et al. 2013). This study provided additional characterization of clicks and whistles from free‐ranging individuals in Xiamen Bay, which was less addressed in previous studies. Our analyses showed that the clicks produced by these dolphins shared general spectral and temporal properties with those reported for the same species and other whistling dolphins (Akamatsu et al. 1998; Fang et al. 2015). The peak frequency of clicks in this study was 86.4 ± 19.5 kHz, which was considerably lower than the values reported in previous studies: 109.0 ± 4.1 kHz for other wild dolphins, 114.1 ± 9.6 kHz for a captive young dolphin, and 97.6 ± 16.4 kHz for a captive older dolphin (Fang et al. 2015; Li et al. 2013). In contrast, the −3 dB bandwidth of 53.3 ± 14.6 kHz in the current paper was higher than 50.3 ± 13.3 kHz, 41.8 ± 14.3 kHz and 47.2 ± 18.7 kHz, respectively, as reported by Li et al. (2013) and Fang et al. (2015). Closely aligned with 22 ± 4 μs of the wild dolphins in Sanniang Bay, the duration of clicks in the current study (22.3 ± 6.4 μs) was lower than those of the two captive individuals (23.0 ± 4.2 μs and 24.7 ± 5.0 μs). This intermediate range may reflect intraspecific diversity due to the geographical and environmental conditions as echolocation click frequencies are known to vary significantly with task demands, behavior, and acoustic environment in other delphinids (Au et al. 1985; Ibsen et al. 2007; Moore and Pawloski 1990). Although such factors likely differed between our study site and the PRE, their specific influences could not be quantified due to limited observational data.

Constant‐frequency whistles were the most common type in the Xiamen population, consistent with observations in PRE, LZB, and SNB populations (Yuan et al. 2021). However, whistles from Xiamen Bay differed in several acoustic parameters from those of other populations. Mean minimum frequencies were 5.2 ± 2.2 kHz (Xiamen Bay), 4.19 ± 2.63 kHz (PRE), 4.45 ± 2.00 kHz (LZB), and 6.35 ± 4.29 kHz (SNB), while the mean maximum frequencies were 7.5 ± 2.7 kHz, 6.61 ± 3.75 kHz, 6.68 ± 2.90 kHz, and 9.49 ± 5.28 kHz, respectively. Population‐specific averages of whistle durations were 247.6 ± 174.2 ms, 461 ± 420 ms, 447 ± 313 ms, and 462 ± 410 ms in Xiamen Bay, PRE, LZB, and SNB, respectively, indicating that whistles from Xiamen Bay were consistently shorter than those from other regions. Whistles of SNB dolphins consistently exhibited higher frequency parameters than those of the populations in Xiamen Bay, PRE, and LZB. Yuan et al. (2021) demonstrated that the acoustic divergence between PRE and LZB was markedly smaller than their respective differences with SNB. These geographic variations in vocal signatures likely reflect the effects of spatial separation and geographic barriers, in agreement with known bioacoustic differentiation patterns in cetaceans (May‐Collado and Wartzok 2008). It has been suggested that the greater spatial separation of 449.8 km between LZB and SNB may function as a substantial biogeographic barrier, contributing to larger differences in whistle parameters compared to those between LZB and PRE (Yuan et al. 2021).

Clicks from the West sub‐region population exhibited shorter durations, higher peak frequencies, and broader bandwidths, which may reflect adaptation to local environmental conditions. The potential acoustic divergence in echolocation signals aligns with the findings of Wang et al. (2015), which defined the population as geographically, behaviorally, and statistically distinct communities. The East sub‐region consists mainly of shallow flats with a sandy substrate and extensive marine aquaculture areas, whereas the West sub‐region also has high productivity but is instead dominated by estuarine processes (turbid, freshwater outflow from the Jiulong River) (Wang et al. 2015). The divergent acoustic features likely represent an adaptive strategy to the more complex and turbid estuarine soundscape, where shorter clicks enhance temporal resolution for close‐range echolocation in turbid waters, broader bandwidths facilitate target discrimination among cluttered substrates, and higher frequencies reduce masking from prevalent low‐frequency ambient noise.

**FIGURE 8:**
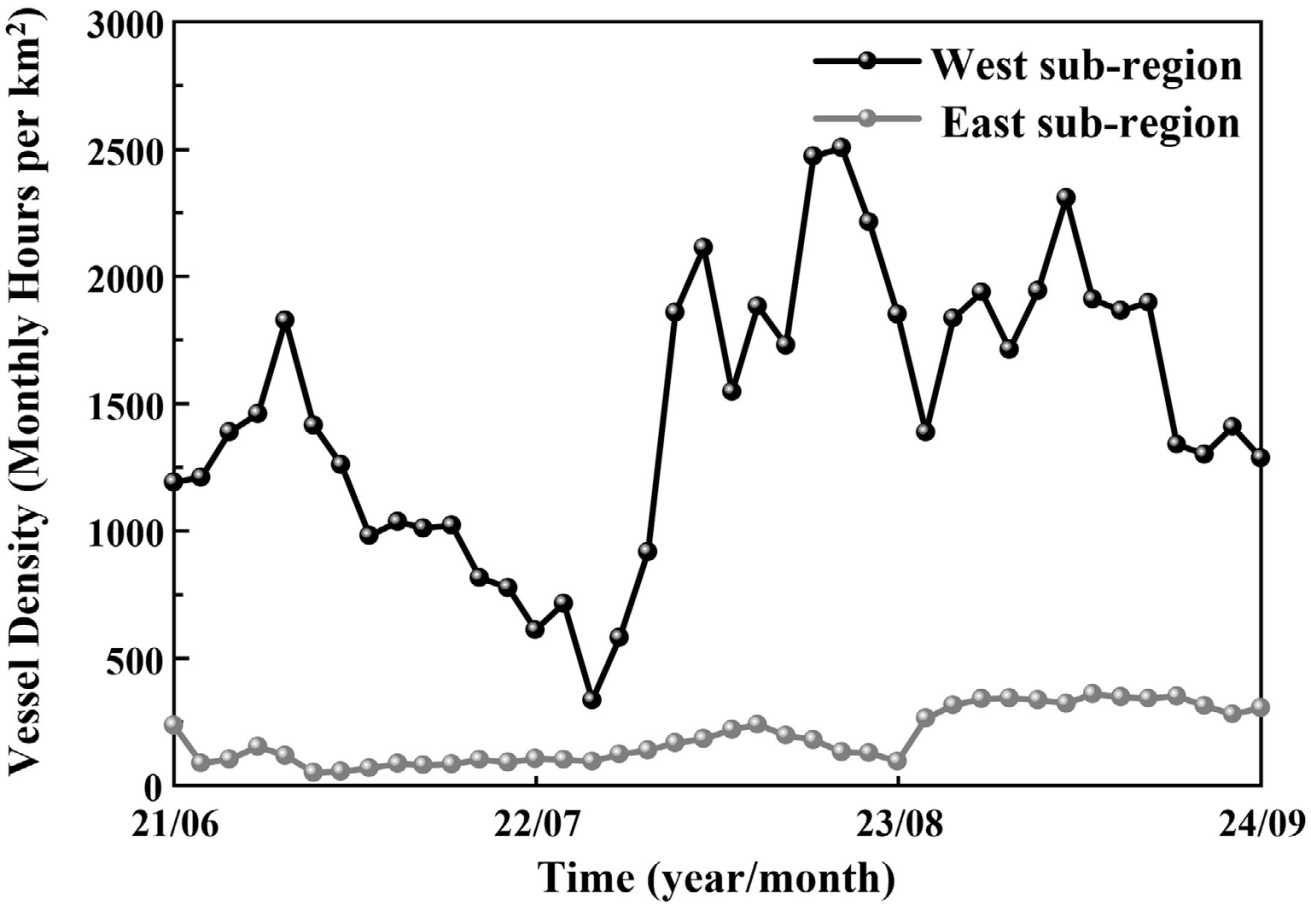
Time series of the vessel density variation from June 2021 to September 2024 in the East and West sub‐regions. The vessel traffic data were obtained from Global Maritime Traffic.

In addition to spatial separation and natural environmental differences, anthropogenic activities, particularly differential vessel traffic, may contribute to the observed variations in click signals. From June 2021 to September 2024, the West and East sub‐regions experienced markedly different levels of vessel interference (Figure 8). Average vessel density in the West sub‐region was 1471.4 ± 538.6 monthly hours per km^2^, significantly higher than 190.3 ± 105.0 h per km^2^ in the East (U = 1593, *p* < 0.01). The shorter duration, higher peak frequency, and broader bandwidth of clicks in the West sub‐region may therefore in part reflect adaptation to busier vessel traffic. Similarly, Heiler et al. (2016) suggested the presence of tour boats would elicit a significant upward shift in frequencies of sound signals produced by the common bottlenose dolphins (*Tursiops truncatus*) in Walvis Bay, Namibia. The comparable phenomenon has also been reported in a resident common bottlenose dolphin of the Cres‐Lošinj archipelago (Rako‐Gospić and Picciulin 2016), and the species in Tampa Bay (Van Ginkel et al. 2018). This frequency increase reflects an active vocal adaptation to boat noise, potentially to enhance signal detectability in noisy environments.

Numerous species, such as beluga whales (*Delphinapterus leucas*) and common bottlenose dolphins, have exhibited the Lombard response, increasing signal amplitude in response to elevated ambient noise (Brumm and Zollinger 2011; Lombard 1911; Scheifele et al. 2005). In the current study, although peak‐to‐peak sound pressure levels of clicks in the West sub‐region were significantly higher, the relationship of source levels between the two sub‐regions remained uncertain due to the lack of exact distance measurements. Further research is needed to quantify vessel‐induced effects on click amplitude under varying noise conditions.

Rako‐Gospić and Picciulin (2016) reported that dolphins increased whistle frequencies in response to elevated low‐frequency noise, whereas exposure to high‐frequency noise led to reductions in maximum, delta, and beginning frequencies, as well as decreased frequency modulation. This indicates that noise adaptation strategies integrate both environmental acoustic conditions and immediate behavioral priorities. In the present study, no significant difference was found in whistle characteristics (*p* > 0.05). It may be partly attributed to the limited dataset, as the total number of whistles was 107. Additionally, although the dolphins in Xiamen Bay were divided into two geographically and statistically distinct communities, a low level of intercommunity interaction was still observed (Wang et al. 2015). Unlike echolocation clicks, which appear to be environmentally driven, whistles serve as primary signals for social communication and group cohesion. Thus, the existence of social connectivity, even if limited, may also contribute to the similarity in whistle features. Previous studies have also shown that whistle similarity was related to the strength of individual social relationships, with closely associated males sharing more whistle types than nonassociated animals (Smolker and Pepper 1999; Watwood et al. 2004). In addition, a similar frequency shift in whistles between social groups has been detected in bottlenose dolphins during the behavioral states characterized by a high level of arousal, such as foraging and socializing (Hawkins 2010; King and Janik 2015; La Manna et al. 2013, 2020; May‐Collado and Quiñones‐Lebrón 2014).

Van Parijs and Corkeron (2001) found that Indo‐Pacific humpback dolphins in eastern Australia produced 17 types of whistles, totaling over 200 whistles in 7 days of recording. Whistling events are more frequent in larger populations, such as those in the Pearl River Estuary (PRE), Leizhou Bei (LZB), and Sanniang Bay (SNB) (Chen et al. 2018; Yuan et al. 2021). The Xiamen population, however, comprises only 54 individuals (Wu et al. 2025; Zhong 2021), substantially smaller than other populations (Chen et al. 2018), which likely limited social interactions and resulted in fewer recorded whistles over the 43 recording days. The geographical segregation and limited intercommunity exchange, involving only nine shared individuals between the West and East communities (Wang et al. 2015), may further reduce whistling activity. In addition, the intermittent sampling remains an inherent limitation of the boat‐based sampling method, which may have led to limited opportunities to collect whistles.

More than one factor needs to be considered when addressing the changes in vocalizations in the Indo‐Pacific humpback dolphins, including group size, boat traffic, coastal formation, and the proportion between adult and young dolphins. Systematic investigation of these factors is needed to better understand their effects on population divergence and acoustic communication. Among them, anthropogenic activities and the associated noise have increasingly emerged as key drivers of vocal behavior changes. Noise has induced considerable impacts on dolphins including behavioral disruptions (Duarte et al. 2021; Erbe et al. 2019; Slabbekoorn et al. 2018), communication masking (Wang, Wu, et al. 2014), and temporary or permanent hearing loss (Southall et al. 2019). Mitigating noise pollution is therefore critical for protecting local Indo‐Pacific humpback dolphins, whose habitats overlap extensively with human‐impacted waters.

Previous conservation management in Xiamen Bay has generally treated the population as a single management unit (Chen et al. 2008, 2009; Huang and Liu 2000; Wang et al. 2015). While consistent with a single population model, the observed acoustic variation in clicks indicates a risk of population fragmentation. Given Xiamen Bay's small population size (Chen et al. 2018; Wu et al. 2025; Zeng et al. 2020) and the potential habitat fragmentation between the West and East sub‐regions (Lu et al. 2024; Zhong 2021), this vulnerable population warrants prioritized conservation attention.

## 5 Conclusions

Over 3 years of field surveys, we recorded vocalizations of Indo‐Pacific humpback dolphins in Xiamen Bay during 43 encounters. The subsequent analysis showed a peak frequency of 86.4 ± 19.5 kHz, −3 dB bandwidth of 53.3 ± 14.6 kHz and duration of 22.3 ± 6.4 μs for 33,038 clicks. Whistles had a frequency range of 2.4 ± 2.1 kHz, minimum frequency of 5.2 ± 2.2 kHz, maximum frequency of 7.5 ± 2.7 kHz and duration of 247.6 ± 174.2 ms. No significant difference was found in characteristics between whistles produced by the West and East groups. However, dolphins in the West sub‐region tended to emit clicks with higher peak frequencies, broader bandwidths and shorter durations, suggesting echolocation adaptations to local natural environmental conditions and vessel noise. These findings indicate the emergence of variation in vocal characteristics between the two communities, highlighting the necessity of establishing additional ecological corridors to facilitate inter‐community exchange, thereby mitigating the risks of population fragmentation and even local extinction. Furthermore, strict vessel noise mitigation strategies are essential to protect this small population.

## Author Contributions

**Xuming Peng:** conceptualization (equal), data curation (equal), investigation (equal), methodology (equal), software (equal), validation (equal), visualization (equal), writing – original draft (equal). **Zhiyuan Hua:** formal analysis (equal), methodology (equal), project administration (equal), validation (equal), visualization (equal). **Fuxing Wu:** conceptualization (equal), funding acquisition (equal), resources (equal), supervision (equal), writing – review and editing (equal). **Fei Zhang:** data curation (equal), investigation (equal), project administration (equal), visualization (equal). **Weijie Fu:** data curation (equal), investigation (equal), methodology (equal), software (equal). **Yupeng Li:** data curation (equal), formal analysis (equal), investigation (equal), project administration (equal). **Chuang Zhang:** data curation (equal), formal analysis (equal), investigation (equal), visualization (equal). **Wenzhan Ou:** formal analysis (equal), investigation (equal), methodology (equal), project administration (equal). **Wenjie Xiang:** data curation (equal), methodology (equal), software (equal). **Bing Zhou:** investigation (equal), project administration (equal), visualization (equal). **Zhongchang Song:** conceptualization (equal), funding acquisition (equal), methodology (equal), resources (equal), supervision (equal), writing – review and editing (equal). **Yu Zhang:** project administration (equal), supervision (equal).

## Funding

This work was funded by the National Natural Science Foundation of China (No. 62231011), the marine ecological early warning and monitoring Foundation of the Ministry of Natural Resources (No. S‐HR04‐230701‐24), the Natural Science Foundation of Fujian Province of China (No. 2024J01019), the Fundamental Research Funds for the Central Universities (No. 20720240106), the National Key R&D Program of China (No. 2024YFD2401402), the Open Fund Project of Hanjiang National Laboratory (No. KF2024030), and the fund (No. Pilab2409) from the State key laboratory of precision measuring technology and instruments (Tianjin University).

## Conflicts of Interest

The authors declare no conflicts of interest.

## Data Availability

The data that supports the findings of this study are available at https://doi.org/10.5281/zenodo.18300177.
